# Interaction specificity between leaf-cutting ants and vertically transmitted *Pseudonocardia* bacteria

**DOI:** 10.1186/s12862-015-0308-2

**Published:** 2015-02-25

**Authors:** Sandra B Andersen, Sze Huei Yek, David R Nash, Jacobus J Boomsma

**Affiliations:** Centre for Social Evolution, Department of Biology, University of Copenhagen, Universitetsparken 15, 2100, Copenhagen, Denmark; Current address: Novo Nordisk Foundation Center for Biosustainability, Technical University of Denmark, Hørsholm, Denmark; Current address: Department of Genetics, Forestry and Agricultural Biotechnology Institute (FABI), University of Pretoria, Pretoria, South Africa

**Keywords:** Attine ant mutualism, Cross-fostering, Prophylactic defences, Host-symbiont coevolution

## Abstract

**Background:**

The obligate mutualism between fungus-growing ants and microbial symbionts offers excellent opportunities to study the specificity and stability of multi-species interactions. In addition to cultivating fungus gardens, these ants have domesticated actinomycete bacteria to defend gardens against the fungal parasite *Escovopsis* and possibly other pathogens. Panamanian *Acromyrmex echinatior* leaf-cutting ants primarily associate with actinomycetes of the genus *Pseudonocardia*. Colonies are inoculated with one of two vertically transmitted phylotypes (Ps1 or Ps2), and maintain the same phylotype over their lifetime. We performed a cross-fostering experiment to test whether co-adaptations between ants and bacterial phylotypes have evolved, and how this affects bacterial growth and ant prophylactic behavior after infection with *Escovopsis*.

**Results:**

We show that *Pseudonocardia* readily colonized ants irrespective of their colony of origin, but that the Ps2 phylotype, which was previously shown to be better able to maintain its monocultural integrity after workers became foragers than Ps1, reached a higher final cover when grown on its native host than on alternative hosts. The frequencies of major grooming and weeding behaviors co-varied with symbiont/host combinations, showing that ant behavior also was affected when cuticular actinomycete phylotypes were swapped.

**Conclusion:**

These results show that the interactions between leaf-cutting ants and *Pseudonocardia* bear signatures of mutual co-adaptation within a single ant population.

**Electronic supplementary material:**

The online version of this article (doi:10.1186/s12862-015-0308-2) contains supplementary material, which is available to authorized users.

## Background

Symbiotic interactions between species can range from mutualistic to parasitic, depending on the levels of cooperation and conflict expressed between the interactors [1,2]. Rarely is the association limited to just two species, so understanding interaction networks is paramount for accurate interpretation of ecologically and clinically important assemblies of organisms e.g. [3-7]. The complex mutualism of the fungus-growing ants has become a model for testing the mechanisms of multi-partite interaction stability and specificity e.g. [8-11]. The ants farm fungal cultivars in clonal monocultures [12-14] and have achieved substantial evolutionary radiation since the symbiosis evolved *ca*. 50 million years ago [15].

Over evolutionary time, a number of other organisms have become associated with the symbiosis, as commensals [16], parasites, additional mutualists, or parasites of additional mutualists [17-19]. In particular, studies have focused on the hypocrealean fungal genus *Escovopsis*, which are specialized ascomycete parasites of the attine cultivars [20,21]. While devastating if uncontrolled, many attine ants harbor antibiotic-producing actinomycete bacteria on their cuticle to protect their gardens with a range of associated behaviors [22-24], but the association specificity between these bacteria, the ants that carry them, and the pathogens they target has been subject to debate [25-33].

*Atta* and *Acromyrmex* leaf-cutting ants are the most evolutionarily derived fungus-growing ants. Both genera harvest fresh plant material to provision their fungus gardens, but only *Acromyrmex* has maintained the ancestral attine habit of growing filamentous actinomycete bacteria on the cuticle of large- and medium-sized workers [17,22,34]. The bacterial cover may have multiple functions; it has been shown to provide a barrier against entomopathogens for newly eclosed (callow) workers [35] but experimental work has primarily focused on its role in defending gardens against *Escovopsis* during the first weeks of callow life, when cuticular actinomycete cover is high and ant behavior is focused on fungus garden maintenance [10,36]. In the two best studied species, *A. echinatior* and *A. octospinosus*, *Pseudonocardia* bacteria have been shown to dominate cuticular communities with both non-specific 454 16S rDNA amplification and culture-based approaches [33,37]. The bacterial cover of large workers follows a characteristic ontogenetic pattern starting with inoculation shortly after eclosion [34], culminating with substantial body cover after two to three weeks, and declining to a modest cover by the time workers become foragers about seven weeks after eclosing.

The initial exponential growth phase towards full cuticular actinomycete cover has been suggested to be supported by nutrients on the ant cuticle and metapleural gland secretions [38], whereas the propleural plates of large workers maintain actinomycete cultures much longer, likely using secretions from specific sub-cuticular glands [22]. *Acromyrmex* colonies have been observed to induce bacterial growth when faced with an experimental *Escovopsis* challenge [10], but the extent to which the host can control bacterial growth, how this may be achieved and whether the performed grooming behaviors depend on the host/symbiont match is not well understood. Answering these questions is therefore important for evaluating the specificity and coevolutionary potential of this mutualistic interaction, which may represent the oldest example of biological control of crop-pests.

The *A. echinatior* population in Gamboa, Panama is well studied, with two phylotypes of *Pseudonocardia* known to be present and colonies maintaining their original phylotype for more than ten years after transfer to the laboratory [33,37]. We use the term “phylotype” after identifying the *Pseudonocardia* symbionts by partial sequencing of the 16S rDNA and EF-1α genes [33,37]. However, while the default transmission route of *Pseudonocardia* is vertical, both across generations of colonies and among workers within colonies [17,33], other actinomycetes than *Pseudonocardia* have also been isolated, both from *Acromyrmex* and other attine ants, suggesting that additional environmental acquisition is common [29,31,32,39,40]. A recent hypothesis proposed by Scheuring & Yu [41] suggests that such diversity may be stable if the initial cuticular community is dominated by a single mutualistic actinomycete phylotype, which would be the case when native vertically transmitted phylotypes are the first to colonize ants after they eclose, consistent with recent 16S rDNA amplicon sequencing results [33].

The aim of the present study was to test whether interaction-specificity of *Acromyrmex* and *Pseudonocardia* bacteria varies among sympatric colonies maintaining different vertically transmitted phylotypes. We designed a cross-fostering experiment to manipulate the first inoculations of eclosing large workers and to monitor the consequences of *Pseudonocardia* swaps for the expression of prophylactic behavioral responses by the ants after infection with *Escovopsis*. When ant pupae are cross-fostered in a different colony than their own, the callow adults that eclose from them readily obtain a bacterial cover, but a previous study indicated that cross-inoculated bacteria reach lower abundance on mature ants three weeks later than they would have on ants reared in their native colony [42]. This suggested that there is some degree of specificity in the association between *Acromyrmex* host colonies and phylotypes of actinomycete symbionts. Such specificity would be consistent with co-adaptation between *Acromyrmex* hosts and actinomycete symbionts within a specific population of ant colonies, whereas recent debate has primarily addressed the dynamics of the attine ant-*Pseudonocardia* mutualism at the species level [26,30,43-45].

**Figure 1:**
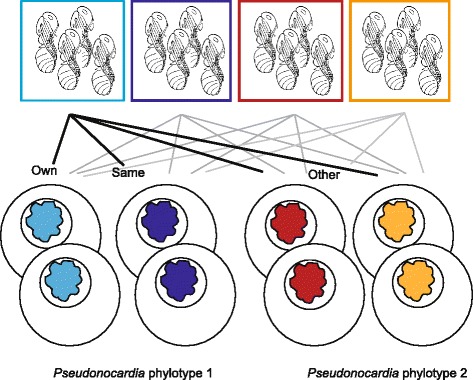
**The cross-fostering experiment set-up.** Fungus gardens and pupae from four source colonies were used to set up 32 subcolonies in a duplicated (4x4) fully crossed design. Source colony identity is indicated by color, with blue used for colonies with *Pseudonocardia* phylotype 1 (Ps1) and red/orange for colonies with *Pseudonocardia* phylotype 2 (Ps2). Pupae from each source colony eclosed in two replicate subcolonies with either a garden fragment and nurse workers from their own colony (*e.g.* light blue pupae in light blue garden), or a garden fragment and nurse workers from a colony with the same *Pseudonocardia* phylotype (*e.g.* light blue pupae in dark blue garden), or a garden fragment and nurse workers from two colonies with the other *Pseudonocardia* phylotype (*e.g.* light blue pupae in red and orange gardens). Drawing of pupae from http://etc.usf.edu/clipart.

We used four *A. echinatior* source colonies, each harboring one of the two previously identified *Pseudonocardia* phylotypes Ps1 and Ps2 [33], to create a fully crossed experimental design of subcolonies where pupae of large workers were raised in an environment with either their own *Pseudonocardia* phylotype or the other phylotype (Figure 1). The bacterial growth rates on the ant cuticles were measured to test whether interaction-specificity constrains bacterial inoculations that are not strictly vertical. In addition, we monitored behavioral reactions of the ants after controlled exposure to *Escovopsis* to test whether horizontally acquired phylotypes would affect prophylactic disease defense behaviors and thus potentially the survival and reproductive success of the ants and their fungus gardens, even though the swapped *Pseudonocardia* phylotypes are native mutualists in other sympatric colonies of the same host ant species. Superior symbiont growth or enhanced behavioral defenses against *Escovopsis* in the presence of a native bacterial symbiont would suggest that ant- and bacterial lineages co-adapt in spite of substantial gene-flow across ant generations, whereas not finding such patterns would be evidence for the absence of variation in interaction-specificity, and thus for much lower potential for co-adaptive evolutionary change.

Our results confirm earlier observations that eclosing callow workers can be successfully inoculated by genetically different *Pseudonocardia* phylotypes from sympatric conspecific colonies, and we found no evidence for differences in host/symbiont specificity at this stage. However, we found consistent differences in final cover across actinomycete phylotypes and, after infecting experimental subcolonies with *Escovopsis*, multiple differences in ant grooming behavior depending on whether they were associated with one or the other *Pseudonocardia* phylotype.

## Results and discussion

### Differential growth rates of *Pseudonocardia* phylotypes on cross-fostered workers

**Figure 2:**
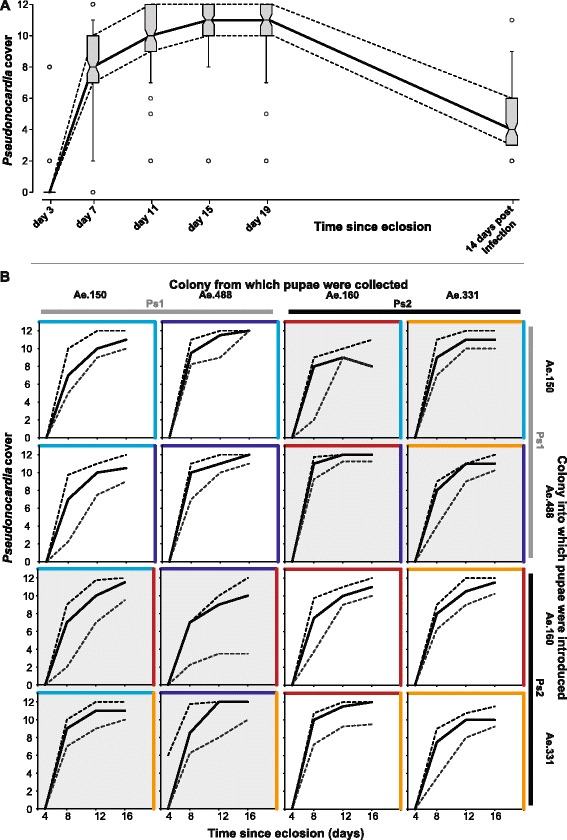
**Bacterial growth rates on ant cuticles. A.** Boxplot of the bacterial cover scores of all individuals from all subcolonies from day 0 – 19 and 2 weeks after *Escovopsis* infection: Box-and-whisker plots, with the middle band representing the median, the bottom and top of the boxes representing the 25th and 75th percentiles, the lower and upper whiskers the 5% and 95% percentiles, and points beyond these shown as open circles. At day 0 all individuals had a score of 0. **B.** Bacterial cover scores with the solid lines showing the median cover and the dashed lines indicating the 25% and 75% percentiles for all individuals in the two replicate subcolonies for each treatment. For each plot the source colony of the fungus garden and the pupae is indicated, in addition to the identity of the native *Pseudonocardia* phylotype (colors as in figure 1). White background plots are crossings of the same phylotype inoculating ants of another colony having the same *Pseudonocardia*, and shaded plots are crossings where gardens/nurses and pupae were from colonies with a different *Pseudonocardia* phylotype.

We confirmed by PCR that the ants after eclosion readily obtained the *Pseudonocardia* phylotype of the fungus garden and the nursing workers they were exposed to, and that both phylotypes showed exponential growth on the ant cuticles during the two weeks following inoculation with no effect of the cross-fostering (Two-way ANOVA on Box-Cox transformed growth rates; phylotype pupae: F_1,94_ = 1.167, p = 0.283, phylotype garden: F_1,94_ = 0.036, p = 0.851, phylotype pupae × garden: F_1,94_ = 0.205, p = 0.652). However, ants from one colony (Ae.488) and some of the combinations of pupae and garden/nurses reached maximum bacterial cover faster than others, consistent with earlier findings (Pupae ID × phylotype pupae; F_2,94_ = 8.001, p < 0.001; ID refers to the source colony number; Figure 2B; Additional file 1: Table S1 [34,38]). Our results thus indicate that there were intrinsic differences between colonies in bacterial growth rate, but that these conditions by themselves were not inducing exclusive forms of *Acromyrmex*-*Pseudonocardia* interaction specificity. A relatively high overall variation in cuticular *Pseudonocardia*-cover between individual ants suggests that uncontrolled factors also affect bacterial growth, such as the bacterial cover of inoculating ants in the fungus garden, potentially including taxa other than *Pseudonocardia* for older workers, and differences in the extent to which standardized laboratory provisioning of colonies may affect genetically different ants and fungus gardens. The fact that *Acromyrmex* pupae are variably covered in hyphae of the garden fungus prior to eclosion [46] might also have affected the conditions for subsequent bacterial growth after eclosing.

**Figure 3:**
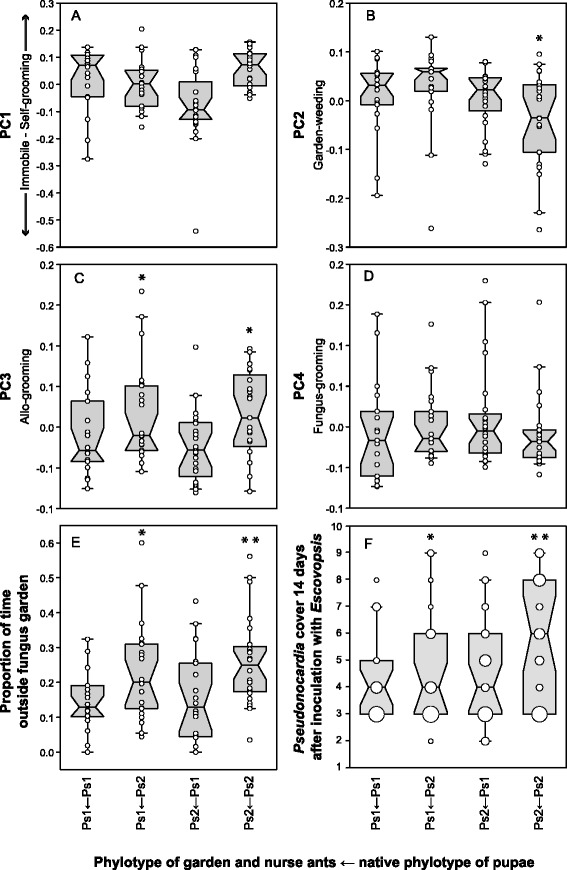
**The effect of** ***Pseudonocardia*** **phylotype combination (in subcolonies composed of pupae and gardens/nurses with Ps1 or Ps2 in all four combinations) on behavioral profiles, usually defined as principal components (A - E, see text for details) and final cover (F).** Box plots are drawn as in figure 2A. **A.** PC1 capturing the negatively correlated behaviors self-grooming (positive values) and immobile in the fungus garden (negative values). Workers with their native phylotype spent more time self-grooming, although this was not statistically significant after Bonferroni correction. **B.** PC2 showing fungus-grooming. Workers with Ps2 as their native phylotype, marked with an *, spend significantly less time fungus-grooming in Ps2 gardens than all others. **C.** PC3 showing allo-grooming. Workers with Ps2 as their native phylotype, marked with an *, spend significantly more time allo-grooming than all others. **D.** PC4 showing garden-weeding. There were no significant differences between phylotypes or their combinations. **E.** Time spent outside the fungus garden. Workers with Ps2 as their native phylotype, marked with an *, spend significantly more time outside. In addition to this overall difference, when they were reared in a Ps1 garden they spent significantly less time outside compared to when reared in a Ps2 garden, marked with **, but still more than workers with Ps1 as their native phyloptype. **F.** Final bacterial cover of cross-fostered ants estimated two weeks after infection with *Escovopsis*. There was a significant effect of *Pseudonocardia* phylotype and an interaction between pupal phylotype and fungus garden phylotype, so workers with Ps2 as their native phylotype reached a higher cover, marked with an *, especially in Ps2 gardens, marked with **. The size of the data points indicate the frequency of the cover index.

A previous study [42] showed that there was a significant effect of source colony ID and cross-fostering on the final bacterial cover of mature *A. echinatior* workers from colonies of the same Panamanian population, which normally have regressed to a coverage score of 2 or 3 (bacterial cover limited to the propleural plates) by the time they become foragers [34]. The effect was subtle but significant because of the large sample size of >900 ants, but with only two source colonies it was not possible to infer whether this effect was colony- or *Pseudonocardia*-phylotype-specific. Our present results confirm by PCR that the colonies used by Armitage *et al*. [42] harbored different phylotypes, Ps1 in Ae.227 and Ps2 in Ae.266, so the results of both studies indicate that cross fostered pupae from a Ps2 colony ended up as mature workers with lower covers when they were inoculated with Ps1, whereas the reduction of Ps1 workers inoculated with Ps2 was much less pronounced (Ordinal logistic model, phylotype pupae: Likelihood-Ratio (L-R) *χ*^2^ = 4.504, df = 1, p = 0.034; phylotype pupae × phylotype garden: L-R *χ*^2^ = 6.299, df = 1, p = 0.012; Figure 3F, Additional file 1: Table S2). We also found significant effects of pupal origin and garden colony ID, with pupae and gardens from Ae.160 (phylotype Ps2) supporting lower final covers than gardens from Ae.331 (Garden ID × garden phylotype: L-R *χ*^2^ = 39.416, df = 2, p < 0.001; pupae ID × phylotype pupae: L-R *χ*^2^ = 10.407, df = 2, p = 0.006). Our estimates of final actinomycete-cover were somewhat higher (median score 4, 25-75% quartiles 3–6) than in Armitage *et al*. [42], consistent with our ants not quite having reached their final low cover typical of foragers by the time we terminated our experiment (they were measured *ca*. 1 week earlier than in [42]). Armitage *et al*. also found that Ps2 ants maintain a higher cover than Ps1 ants as they develop towards becoming foragers (Figure 3F).

The combined results from both studies thus support the idea that there are *Pseudonocardia* phylotype-specific effects on the final bacterial cover of large *Acromyrmex* workers, with one of the phylotypes maintaining a higher cover when grown on its native host, consistent with some degree of co-adaptation and host-specificity, potentially mediated by secretions from the subcuticular glands under the propleural plates [22]. The results recently obtained by Andersen *et al*. [33] showed that older (foraging) workers from Ps1 colonies acquire a more diverse cuticular community (still consisting mostly of actinomycetes), whereas older workers from Ps2 colonies retain their monocultures almost unchanged. Whether these novel acquisitions grow in competition with Ps1 remains to be confirmed but their relative abundances as documented by Andersen *et al*. [33] indicated that this was likely the case. However, the extent and diversity of the bacterial cover for workers that have reached the foraging stage is unlikely to be of importance in fungus garden disease-defense and transmission of *Pseudonocardia* to newly eclosed workers [47], as these foragers are no longer nurses.

### Behavioral changes induced by cross-fostering

Our cross-fostering approach allowed us to explicitly analyze whether subtle forms of co-adaptation between ant host colonies and their *Pseudonocardia* symbionts are likely to have shaped prophylactic behavior following *Escovopsis* exposure. As we detail below, the Ps1 and Ps2 phylotypes, which occur in *ca.* 50/50 monocultures across the *A. echinatior* population in Gamboa [33,37] appear to have co-adapted to some extent to the maternal ant lineages that maintain them by vertical transmission, in spite of panmictic gene flow *via* the multiple males that inseminate queens every generation. One of the four behavioral profiles that we describe below is significantly different between workers with different native pupal *Pseudonocardia* phylotypes, and one behavioral profile and the time spent outside the fungus garden are significantly different for ants with different combinations of native pupal phylotype and the phylotype of the colony into which they were introduced.

**Figure 4:**
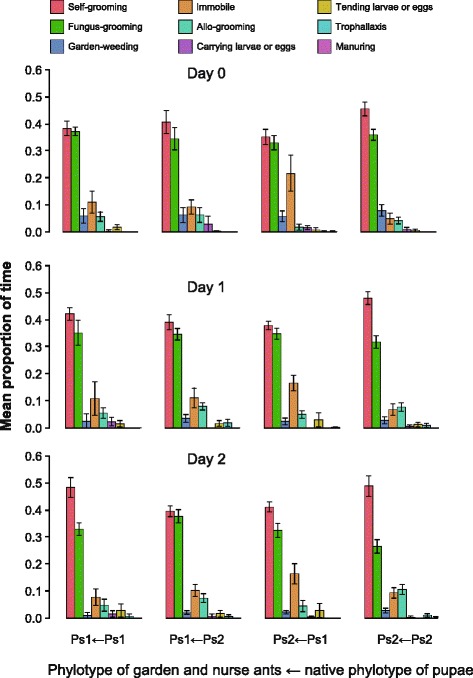
**Nine monitored behaviors expressed by tending large worker ants 15–19 days after eclosion following** ***Escovopsis*** **infection of their gardens, presented as mean proportions of time spent on each behavior ± SE.** Plots are organized according to fostering treatments with garden phylotype followed by pupal phylotype (columns) and time since colonies were infected on Day 0 (rows). Self-grooming, fungus-grooming and allo-grooming were the most common behaviors, whereas garden-weeding was mainly observed on the day of infection (Day 0) and decreased significantly over the three days.

Callow workers with extensive bacterial cover have been suggested to be particularly important for defense against *Escovopsis* in the fungus garden [10]. Vectoring the bacterial antibiotics towards their disease targets should be closely connected to the expression of adequate grooming and weeding behaviors known to be important for the successful control of *Escovopsis* infections [48]. Large callow *Acromyrmex* workers with a full *Pseudonocardia* cover have been found to often sit in the lower parts of fungus gardens, where infection problems with *Escovopsis* are most likely to occur [49]. On average 80% of the behaviors that we observed were performed in the fungus garden and we summarize here only the significant main effects and interaction terms, referring the reader to Additional file 1: Table S3-6 for a complete overview. Self- and fungus-grooming dominated among the garden behaviors, consistent with callow workers having exclusive indoor tasks, and fungus-grooming being particularly positively correlated with bacterial cover (Nested ANCOVA, F_1,66_ = 7.890, p = 0.007, Figure 4; Additional file 1: Table S4). In all instances, fungus-grooming preceded garden-weeding (Currie and Stuart [48]), but only the frequency of garden-weeding decreased significantly over the three days of our experiment (Nested ANCOVA, F_2,66_ = 7.629, p = 0.001, Figure 4), suggesting that this particular behavior (mostly by Ps2 workers) reduces the acute *Escovopsis* threat, whereas fungus-grooming and allo-grooming (mostly by Ps1 workers) represent ongoing routine maintenance of fungus gardens.

Of the nine ant behaviors recorded in infected fungus gardens, the ants spent most of their time self-grooming and fungus-grooming. Self-grooming was performed less by ants with a non-native symbiont, particularly Ps1 ants receiving a cross fostered inoculation with Ps2 (Nested ANCOVA, phylotype pupae × phylotype garden: F_1,66_ = 4.739, p = 0.033, Figure 3A, Additional file 1: Table S3), although this did not retain significance after Bonferroni correction (threshold αʹ = 0.0125). If self-grooming serves a function in the maintenance of cuticular bacteria, this is consistent with ants that were forced to work with a non-native *Pseudonocardia* phylotype being less motivated to protect their cuticular actinomycetes against secondary invaders, particularly when the new phylotype (Ps2) was more robust against such secondary invasions [33]. We also found that native-Ps2 workers groomed their gardens less frequently (Nested ANCOVA, F_1,66_ = 8.221, p = 0.006, Figure 3B). In contrast, allo-grooming of sisters by native-Ps2 workers occurred at a higher rate than allo-grooming by Ps1 workers (Nested ANCOVA, F_1,66_ = 14.121, p < 0.001, Figure 3C; Additional file 1: Table S5). There were no significant differences between phylotypes or their combinations for PC4, representing garden-weeding (Additional file 1: Table S6).

Finally, there was an increase in the proportion of time the workers spent outside the fungus garden over the three days following *Escovopsis* infection (Ordinal logistic model, L-R *χ*^2^ = 7.047, df = 2, p = 0.030). Relative to native-Ps1 workers, native-Ps2 workers spent more time outside their gardens (L-R *χ*^2^ = 13.194, df = 1, p < 0.001; Figure 3E), especially when tending a Ps2 garden (L-R *χ*^2^ = 7.044, df = 1, p = 0.008, Additional file 1: Table S8). We note, however, that the degree to which *Escovopsis* control was ultimately successful could not be estimated in our experiments, so that detailed interpretations of our results will need further validation.

## Conclusions

Around the turn of the century, the recognition of *Escovopsis* as an omnipresent fungus garden parasite and the discovery of cuticular *Pseudonocardia* actinomycetes as obligate defensive symbionts fundamentally changed our interpretation of the complexity of the attine fungus-growing ant symbiosis [17,20,50]. Later work underlined the broad-scale adaptive nature of the *Pseudonocardia* cuticular symbiosis across the attine phylogenetic tree [8,22,51,52], until coevolutionary interpretations started to attract criticism [26], because attine cuticular actinomycetes were shown to be often taxonomically diverse [28], did not always (exclusively) inhibit *Escovopsis* [25,32], and could be environmentally acquired [53], while still producing antibiotics when growing on the cuticles of attine ants [29,39,40,54]. However, our present study and Andersen *et al*. [33] suggest that scenarios of co-adaptation between *Pseudonocardia* and specific lineages of *Acromyrmex* leaf-cutting ants are valid in *Acromyrmex* leaf-cutting ants.

Our results and those by Poulsen *et al*. [34] and Armitage *et al*. [42] show that different *Pseudonocardia* phylotypes can readily colonize the cuticle of callow large workers of *Acromymex* ants, and we found no indications of any specific host-symbiont recognition or discrimination at this stage. However, such discrimination also seems unlikely to have been selected for, because callow ants are inoculated in the fungus garden in which they eclose and with actinomycetes that are long-term associated with the fungus garden and/or their sister workers [33]. A recently published study has suggested that the opportunity for colonization may be limited to a time frame of just a few days immediately following eclosion and showing that callow workers were only successfully inoculated when in the company of large sister workers with a full bacterial cover [47]. However, sample sizes of that study were very small, so could at best show that likelihoods of inoculation differed, consistent with our present results, which show that callow large workers almost always developed a full bacterial cover in the presence of only small and medium size workers and a fungus garden fragment. Together these findings match predictions from the model by Scheuring & Yu [41], demonstrating that initial colonization with an actinomycete of proven usefulness to the farming symbiosis will guarantee that later secondary acquisitions will not challenge the mutualistic nature of the entire cuticular biofilm, consistent with old foragers of *A. echinatior* never completely losing their native symbiont even though their cuticular biofilm may become dominated by other actinobacteria later in life [33].

Our present study indicates that evolutionary processes of mutual co-adaptation between female lineages of leaf-cutting ant hosts and phylotypes of cuticular *Pseudonocardia* may operate at the within-population level. A promising line of future research may therefore be to investigate whether the Ps1 and Ps2 phylotypes of *A. echinatior* in Gamboa may associate with maternally inherited fungus garden clones and even ant mitochondrial genotypes, and to extend such approaches to sympatric populations of other fungus-growing ants to see whether common patterns of inheritance and lineage sorting may emerge. The different behavioral syndromes of the ant hosts in response to their gardens being challenged with the same *Escovopsis* strain suggests that there may be different ways of being a successful native *Pseudonocardia* symbiont of Panamanian *A. echinatior*, resulting in a mixed evolutionary stable strategy [55] consistent with the ca. 50/50 prevalence of the two phylotypes in the population as a whole.

It will be important to do similar studies with other sympatric *Escovopsis* strains to test the generality of the Ps1- and Ps2-induced defensive behaviors, and to investigate the efficiencies by which Ps1 and Ps2 can control *Escovopsis* infections in combination with the behavioral syndromes of their host ants, as that will ultimately determine whether and how these phylotypes stably coexist in the same population while excluding each other at the level of host colonies. Future studies will also need to study the chemical compounds by which ant colonies, Ps1, and Ps2 interact. Our present results indicate that such studies should retain focus on individual and colony-level variation rather than dismissing co-evolutionary adaptations on the basis of species-level studies only.

## Methods

### Cross-fostering

A cross-fostering experiment with a fully crossed design was set up in a temperature (*ca*. 25°C) and humidity (*ca*. 70%) controlled rearing room, using four source colonies of *Acromyrmex echinatior* with queens collected in Gamboa, Panama between 2001 and 2010. Colonies Ae.150 and Ae.488 harbored the native *Pseudonocardia* phylotype ‘Ps1’, while colonies Ae.160 and Ae.331 had native *Pseudonocardia* phylotype ‘Ps2’ [33]. Replicate subcolonies were created with two grams of fungus garden (including small brood items), 25 small workers, 15 medium workers and 4 mature large worker pupae. Fungus garden fragments were placed in petri dishes (∅ 5.5 cm) with a hole in the side, allowing ants access to a larger petri dish (∅ 9 cm) with moist cotton wool and bramble leaf fragments (renewed regularly) on which to forage. Large worker pupae were distributed such that they eclosed in subcolonies containing fungus and workers from: 1. Their own colony; 2. Another colony with the same *Pseudonocardia* phylotype, and 3. Two colonies harboring the other *Pseudonocardia* phylotype. Each combination was replicated twice resulting in a total of 16 different combinations and 32 subcolonies (Figure 1).

During daily monitoring we marked eclosing large workers with nail-polish between the pronotal spines, using four different colors to make each callow ant individually recognizable [34]. The date of pupal eclosion was recorded as Day 0 and the ventral and dorsal side of the ants were photographed on days 3, 7, 11, 15 and for some individuals also on day 19. The photographs were printed and the bacterial cover scored blindly according to the scale (1–12) of Poulsen *et al*. [34]. However, in our experiments with *A. echinatior*, the bacterial growth did not entirely follow this highly specific sigmoid ontogeny, as bacterial cover on the dorsal side sometimes preceded growth on the propleural plates (these deviating individuals were observed in all treatments), consistent with maximal covers in *A. echinatior* being somewhat less abundant than in *A. octospinosus* [56]. To take into account these inter-species differences, we scored bacterial cover primarily based on dorsal growth [57].

All 128 pupae eclosed into large workers, but nine (7%) died shortly after eclosion. These mortalities were all from the same source colony (Ae.150), were equally distributed between the treatments, and were most likely caused by desiccation of pupae during subcolony setup. All dead workers except one were replaced with new mature pupae. Only one newly eclosed worker from another source colony did not develop properly and was removed from the experiment. Five dead medium workers were also replaced during the experiment.

### Prophylactic behavior following *Escovopsis* exposure

When either all individuals were fully covered in bacteria (scale 12) or all ants were at least 15 days post-eclosion, the fungus gardens were exposed to infection with a single strain of *Escovopsis weberi*. This slight difference in procedure was unavoidable due to differences between pupae within subcolonies in the timing of bacterial growth, and limitations in the number of sub-colonies that could be handled by a single observer in a day. For each treatment, *Escovopsis* conidia were collected with a fine paintbrush from 1 cm^2^ of pure *E. weberi* culture, corresponding to 1.3-2.5 × 10^7^ dry conidia, and transferred to the fungal cultivar by brushing evenly over the fungal surface. The *E. weberi* strain was isolated from an *Acromyrmex* nest in Gamboa, Panama by H. Fernández-Marín in 2010, identified based on morphological characters [58] and grown on potato dextrose agar medium. The *E. weberi* conidia that we used were highly viable as their germination rate on plates was > 90%.

During the first hour after exposure, behavioral repertoires expressed by the four marked workers were observed for 5 min in the 3.5 cm diameter field of view of a stereomicroscope (6.3× magnification) focused on the fungus garden, with 5 min intervals between each observation, resulting in 6 time points for the first hour. Behaviors were recorded in 30s time blocks; if a marked individual was observed to continuously perform the same behavior within a 30s block it was recorded as having performed that behavior once, whereas it was recorded as twice when it performed the behavior in two 30s blocks and so on. Activities that were performed for less than 30s were not recorded. This gave us a quantitative estimate of continuous behaviors per subcolony and meant that less important transient behaviors were not recorded. Behavioral repertoires were recorded again after 48 and 72 hours since exposure, using the same time interval (5 min observation, 5 min break), resulting in 18 windows of 5 min for each subcolony [57]. All behaviors were scored by a single observer who was blind to the received cross-fostering treatment of subcolonies.

Nine different behaviors were recorded after *Escovopsis* exposure of the fungus garden fragments: self-grooming, fungus-grooming, garden-weeding, immobile inside fungus garden, allo-grooming, carrying eggs/larvae/pupae, tending eggs/larvae/pupae, trophallaxis, and garden manuring. We defined grooming behaviors as scraping antennae and legs with forelegs, licking forelegs with mouth parts [59], followed by application of forelegs to different locations. If the target of grooming was a worker’s own body, we categorized it as ‘self-grooming’, if the target was a nestmate, we categorized it as ‘allo-grooming’, and if the target was eggs, larvae or pupae, we categorized it as ‘tending eggs/larvae/pupae’. ‘Fungus-grooming’ was scored when the ants antennated the fungus garden, extended their maxillae and labium to grasp a piece of the garden matrix, and closed them to retract and raise the fungal fragment off the garden matrix while pulling it through their mouth parts [48]. We categorized ‘garden-weeding’ as actions involving the physical removal of garden pieces, whereby the ants detached a piece of fungus garden from the garden matrix by rocking laterally, side to side, while pulling. Once the piece was detached from the garden, the ants picked it up and carried it to the dump [48].

Often, ants stayed motionless for prolonged periods of time hidden under or sometimes on the fungus garden, a behavior that we categorized as “immobile in fungus garden”. ‘Trophallaxis’ was characterized as transfer of liquid between two workers and ‘manuring’ as gaster-curving (bending the abdomen minus the first three segments) followed by production of a fecal droplet, picking it up with the mandibles and depositing it onto the fungus garden [60]. We categorized the physical retrieval and active moving around of eggs/larvae/pupae by the ants as ‘carrying eggs/larvae/pupae’. If marked individuals were outside the fungus garden (and hence out of the field of view) for the whole of any 30s block, we recorded this as “outside fungus garden”.

### Final bacterial cover and PCR analysis of *Pseudonocardia* phylotypes

Following the 3 days of behavioral observations after *Escovopsis* treatment the subcolony fungus garden fragments were replaced with 2 g of fresh fungus garden including *ca*. 25 medium workers and 50 small workers from the same source colony that experimental workers eclosed in, ensuring that the subcolonies had fresh fungus garden and work force to maintain the fungal cultivar. The bacterial cover of each marked worker was estimated two weeks after *Escovopsis* exposure as described above. Subsequently the ants were frozen at −20°C for PCR identification of the *Pseudonocardia* phylotypes.

From each of the subcolonies, one individual ant was used for analysis of *Pseudonocardia* phylotype identity. Samples from colonies Ae.227 and Ae.266 were also included for identification of their native *Pseudonocardia* phylotype, to allow comparison of our present results with the earlier cross-fostering experiment [42]. The propleural plates were dissected off the ants under a stereomicroscope following the procedure outlined in Andersen *et al*. [33] and DNA extracted with the DNEasy Blood and Tissue kit following the manufacturer’s instructions (Invitrogen, Hilden, Germany). Part of the elongation factor EF-1α gene was amplified with the *Pseudonocardia* specific primers 52 F and 920R [37] in 20 μL PCR reactions with the AmpliTaq Gold kit (Applied Biosciences, New Jersey, USA) at the conditions: 95°C for 4 min followed by 40 cycles of 95°C for 30 s, 62°C for 50 s and 72°C for 2 min and final extension at 72°C for 10 min. PCR products were purified with an MSB Spin PCRapace kit (Invitek, Berlin, Germany) and sequenced by Eurofins MWG Operon (Ebersberg, Germany). Successful PCR amplification was achieved for all but three experimental subcolonies that either had a very low bacterial cover or failed for unknown reasons. The sequences were trimmed and compared in Sequencher 4.7 and the consensus sequences of both phylotypes compared to known sequences with a NCBI GenBank BLAST search. These were 99% similar (Ps1, [GenBank: DQ098127]) and identical (Ps2, [GenBank: DQ098133]) to sequences of bacteria sampled in the same area by Poulsen *et al*. [37] and 95% similar to each other.

### Data analyses, bacterial cover

Bacterial cover was estimated for each individual at each time step (t), and a logistic growth equation (Cover = KP_0_e^rt^ / K + P_0_(e^rt^-1)) fitted to the data, with K (the “carrying capacity”) set to the maximum cover (scale 12), and the value of r (the intrinsic rate of increase in bacterial cover) estimated for each individual using iterative least squares fitting executed in the solver add-in of Microsoft Excel 2011. The single value of P_0_ (the bacterial cover at day 0) that provided the best fit to the data across all individuals was estimated iteratively to be 2.67 × 10^−4^. Bacterial cover on Day 19 was only estimated for 21 of the 32 subcolonies, as the remaining were already treated with *Escovopsis* at this stage, but for these subcolonies the bacterial cover was not significantly different from the cover on Day 15 (Wilcoxon Signed-Rank test, S = −7, p = 0.436; Figure 2A).

Growth rates were Box-Cox transformed to maximize normality and homogeneity of variance for analysis. The rates of increase in bacterial cover and the final cover two weeks after infection were analyzed with a two-way ANOVA to test whether *Pseudonocardia* phylotype of the foster garden and nurses, the native phylotype that the pupae should have been inoculated with, and the interaction between the two affected cuticular growth. The ID of the source colony of the fungus gardens and the pupae was included as a nested variable with the replicate subcolonies nested within. Analyses were carried out using JMP 10.0.2 for Macintosh.

The achieved power of the analysis was analyzed post-hoc with G*Power 3.1.4 [61] to see how likely we were to detect a *Pseudonocardia* phylotype effect given the substantial variation between colonies and our sample sizes. This showed that an effect size (f) of around 0.3 could be detected with a power of 80%, which corresponds to a medium effect size in Cohen’s classification [62]. Substantial differences in bacterial growth rate between treatments should thus have been detectable with the sample size used in our study, suggesting that any *Pseudonocardia* phylotype-specific effects are generally smaller than colony-specific effects.

The final bacterial cover on ants two weeks after *Escovopsis* exposure was analyzed with an ordinal logistic model using maximum likelihood in JMP 10.0.2 for Macintosh, to test whether the *Pseudonocardia* phylotype of the foster garden and nurses, the native phylotype that the pupae should have been inoculated with, and the interaction between the two affected it. Three subcolonies lost more than half of their fungus garden in the course of the experiment, but the decreased garden mass did not affect the growth of *Pseudonocardia* cover (One-Way ANOVA, F_1,125_ = 0.142, p = 0.707). However, they maintained a high bacterial cover rather than showing the characteristic decrease of workers from the other subcolonies, and so had a significantly higher cover two weeks after infection (Ordinal logistic model, Likelihood-Ratio *χ*^2^ = 6.50, d.f. = 1, p = 0.011). As a result, these subcolonies were excluded from the analysis of final bacterial cover, and from the behavioral analyses to avoid the possibility that reduced garden mass could affect the results.

### Data Analyses, behavior after *Escovopsis* exposure

Since the different behavioral responses to *Escovopsis* infections were not independent, as only one behavior could be recorded during each 30s period by each worker, and the performance of several behaviors was highly correlated (Additional file 1: Table S7), behavioral responses were converted to behavioral profiles using principal component analysis. Principal components were calculated using covariances, based on the proportion of 30 s periods in which each behavior was performed. The first four principal components (PC1-4) explained 48.0%, 24.0%, 10.5% and 9.8% of the variation in all behaviors respectively, and were judged to capture all (>90%) significant variation in behavior based on a scree-plot and the Jolliffe criterion [63]. PC1 captured the highly negatively correlated behaviors “Self-grooming” (higher values) and “Immobile in fungus garden” (lower values), while PC2 primarily captured “Fungus-grooming”, PC3 “Allo-grooming”, and PC4 “Garden-weeding”. For more details of the PCA analysis see Additional file 2: Figure S1 and Additional file 1: Table S3-S6.

Variation in the first four principal components was examined using ANCOVA with time (days) and phylotype combination of the source colonies of garden/nurse-workers and pupae as main effects, and median *Pseudonocardia* cover as a covariate (Additional file 1: Table S3-6). As above, the ID of the source colony of the fungus garden and the pupae was included as a nested variable with the replicate subcolonies nested within. Due to substantial loss of garden biomass in three subcolonies from source colony Ae.150 at the time of *Escovopsis* exposure, we excluded these from further analyses. Since the analysis of the four principal components represented tests of the same general hypothesis (change in behavior related to *Pseudonocardia* phylotype and colony of origin), table-wise adjustment of significance levels was carried out using Bonferroni correction, setting αʹ to 0.0125. The proportion of time spent outside the fungus garden was analyzed separately using a Generalized Linear Model (GLM) with binomial errors, correcting for overdispersion (Additional file 1: Table S8). Once again the GLM examined the effects of time (days), phylotype combination, median *Pseudonocardia* cover, and their interactions on time spent outside the garden.

### Availability of supporting data

The data sets supporting the results of this article are available in the Dryad repository, [http://www.dx.doi.org/10.5061/dryad.53vg0].

## Additional files

Additional file 1:
**Bold text and green highlighting denote significant effects with p < 0.05 for Table S1, S2 and S8.** The ID of the colony used as the source of fungus garden and pupae were included as nested variables with the replicate subcolonies nested within for Table S1 and S2. **Table S1.** Results of two-way ANOVA of bacterial growth rates, estimated by a logistic growth model, and the phylotype combination of pupae and fungus gardens. **Table S2.** Results of ordinal logistic analysis of bacterial cover 2 weeks after infection, and the phylotype combination of pupae and fungus gardens. We report Likelihood-Ratio (L-R) Chi-squared values, degrees of freedom (df) and p-values of effect tests. **Table S3.** Results of stepwise ANCOVA on the first principal component (PC1) with time (days) and the phylotype combination of pupae and fungus gardens as main effects, and median *Pseudonocardia* cover as a covariate, excluding three subcolonies that had suffered significant garden loss. We report F-ratios, degrees of freedom (df) and p-values of effect tests. Bold text and green highlighting denote significant effects with p < 0.0125 (following table-wise Bonferroni correction). **Table S4.** As Table S3 for PC2. **Table S5.** As Table S3 for PC3. **Table S6.** As Table S3 for PC4. **Table S7.** Correlations matrix of the nine monitored behaviors, excluding three subcolonies that had suffered significant garden loss. Correlations were calculated based on behavioral profiles obtained by Principal Component Analysis, using covariance methods (see methods text for details). **Table S8.** Results of a generalized linear model on the proportion of time spent outside the fungus garden with binomial errors, correcting for over-dispersion, testing the effect of time (days), the phylotype combination of pupae and fungus garden, median *Pseudonocardia* cover and their interactions. We report Likelihood-Ratio (L-R) Chi-squared values, degrees of freedom (df) and p-values of effect tests. Additional file 2: Figure S1. Loading plots of the first four principal components (PC1-4) from a principal component analysis of the nine behaviors monitored within subcolony fungus gardens of our cross-fostering experiment. In each plot, the loading of particular components of behavior is plotted on a scale ranging from −1 to 1. Behaviors with the highest loadings for each PC are shown in bold, with the imputation that variation in those behaviors is well-captured by the principal component.
