# Supplementary material for: Interaction specificity between leaf-cutting ants and vertically transmitted Pseudonocardia bacteria
Source: BMC Evol Biol. 2015 Feb 25;15:27. doi: 10.1186/s12862-015-0308-2 (PMC4346108; doi:10.1186/s12862-015-0308-2)
Supplement: Additional file 1: — Bold text and green highlighting denote significant effects with p < 0.05 for Table S1, S2 and S8. The ID of the colony used as the source of fungus garden and pupae were included as nested variables with the replicate subcolonies nested within for Table S1 and S2. Table S1. Results of two-way ANOVA of bacterial growth rates, estimated by a logistic growth model, and the phylotype combination of pupae and fungus gardens. Table S2. Results of ordinal logistic analysis of bacterial cover 2 weeks after infection, and the phylotype combination of pupae and fungus gardens. We report Likelihood-Ratio (L-R) Chi-squared values, degrees of freedom (df) and p-values of effect tests. Table S3. Results of stepwise ANCOVA on the first principal component (PC1) with time (days) and the phylotype combination of pupae and fungus gardens as main effects, and median Pseudonocardia cover as a covariate, excluding three subcolonies that had suffered significant garden loss. We report F-ratios, degrees of freedom (df) and p-values of effect tests. Bold text and green highlighting denote significant effects with p < 0.0125 (following table-wise Bonferroni correction). Table S4. As Table S3 for PC2. Table S5. As Table S3 for PC3. Table S6. As Table S3 for PC4. Table S7. Correlations matrix of the nine monitored behaviors, excluding three subcolonies that had suffered significant garden loss. Correlations were calculated based on behavioral profiles obtained by Principal Component Analysis, using covariance methods (see methods text for details). Table S8. Results of a generalized linear model on the proportion of time spent outside the fungus garden with binomial errors, correcting for over-dispersion, testing the effect of time (days), the phylotype combination of pupae and fungus garden, median Pseudonocardia cover and their interactions. We report Likelihood-Ratio (L-R) Chi-squared values, degrees of freedom (df) and p-values of effect tests. [file 12862_2015_308_MOESM1_ESM.pdf]

**Supplementary tables showing details of the statistical analysis**

In the following analyses, "phylotype garden" refers to the phylotype of bacteria found on the nurses and the fungus garden, while "phylotype pupae" refers to the phylotype of bacteria found on the cuticle of workers in the colony from which experimental pupae were collected. Similarly "garden colony" refers to the original stock colony from which the subcolony in which the experimental pupae eclosed was derived, and "pupae colony", the original stock colony from which pupae were collected. Nested terms are shown with the terms within which they are nested in square brackets. Significant terms ($p < 0.05$) are shown with p-values marked in bold, followed by an explanantion of the source of the significant difference. Note that for the analysis of behavioural data (Tables 3-6), table-wise Bonferroni correction was applied, such that $p < 0.0125$ was considered significant.

**Table 1: ANOVA, Growth rate of bacterial cover**

| ***Source*** | $df_n$ | $df_d$ | ***F*** | ***p*** | ***notes*** |
|---|---|---|---|---|---|
| phylotype garden | 1 | 94 | 0.0356 | 0.8507 | |
| phylotype pupae | 1 | 94 | 1.1672 | 0.2826 | |
| phylotype garden × phylotype pupae | 1 | 94 | 0.2049 | 0.6518 | |
| garden colony [phylotype garden] | 2 | 94 | 3.9313 | **0.0228** | Workers hatching in gardens from Ae.160 have lower bacterial growth rates than workers hatching in gardens from Ae.331 |
| pupae colony [phylotype pupae] | 2 | 94 | 8.0015 | **0.0006** | Workers from Ae.150 have lower bacterial growth rates than workers from Ae.488 |
| garden × pupae colony [phylotype garden,phylotype pu | 4 | 94 | 1.2089 | 0.312 | |
| replicate [garden,pupae] | 16 | 94 | 1.7251 | 0.0541 | |

**Table 2: Ordinal logistic analysis of bacterial cover score 2 weeks after infection**

| ***Source*** | ***df*** | ***L-R*** $\chi^2$ | ***p*** | ***notes*** |
|---|---|---|---|---|
| phylotype garden | 1 | 0.071 | 0.7900 | |
| phylotype pupae | 1 | 4.504 | **0.0338** | Workers with Ps2 have a higher final cover |
| phylotype garden × phylotype pupae | 1 | 6.299 | **0.0121** | Workers with Ps2 have a higher final cover in gardens with Ps2 |
| garden colony [phylotype garden] | 2 | 39.416 | **<.0001** | Workers in gardens from Ae.160 have lower final cover than workers in gardens from Ae.331 |
| pupae colony [phylotype pupae] | 2 | 10.407 | **0.0055** | Workers from Ae.160 have lower final cover than workers from Ae.331 |
| garden × pupae colony [phylotype garden,phylotype pu | 4 | 5.119 | 0.2753 | |
| replicate [garden,pupae] | 16 | 28.219 | **0.0133** | |

**Table 3: Nested ANCOVA, PC1**

| ***Source*** | $df_n$ | $df_d$ | ***F*** | ***p*** | |
|---|---|---|---|---|---|
| Time | 2 | 66 | 0.3858 | 0.6814 | |
| phylotype garden | 1 | 66 | 0.2581 | 0.6131 | |
| phylotype pupae | 1 | 66 | 9.4042 | **0.0031** | Workers with a non-native phylotype spend less time self-grooming |
| Time × phylotype garden | 2 | 66 | 0.2796 | 0.7570 | |
| Time × phylotype pupae | 2 | 66 | 1.6462 | 0.2006 | |
| phylotype garden × phylotype pupae | 1 | 66 | 4.7385 | 0.0331 | |
| Time × phylotype garden × phylotype pupae | 2 | 66 | 0.0426 | 0.9583 | |
| Median bacterial cover score | 1 | 66 | 0.5660 | 0.4545 | |
| garden colony [phylotype garden] | 2 | 66 | 3.9186 | 0.0247 | |
| pupae colony [phylotype pupae] | 2 | 66 | 3.6302 | 0.0319 | |
| garden × pupae colony [phylotype garden,phylotype pu | 4 | 66 | 2.2515 | 0.0729 | |

**Table 4: Nested ANCOVA, PC2**

| ***Source*** | $df_n$ | $df_d$ | ***F*** | ***p*** | |
|---|---|---|---|---|---|
| Time | 2 | 66 | 2.3238 | 0.1059 | |
| phylotype garden | 1 | 66 | 0.7874 | 0.3781 | |
| phylotype pupae | 1 | 66 | 1.2604 | 0.2656 | |
| Time × phylotype garden | 2 | 66 | 0.5511 | 0.5790 | |
| Time × phylotype pupae | 2 | 66 | 0.2037 | 0.8162 | |
| phylotype garden × phylotype pupae | 1 | 66 | 8.2213 | **0.0056** | Workers with Ps2 as their native phylotype spend less time garden-grooming in their own garden |
| Time × phylotype garden × phylotype pupae | 2 | 66 | 2.7312 | 0.0725 | |
| Median bacterial cover score | 1 | 66 | 7.8899 | **0.0065** | Garden-grooming increases with increasing cover |
| garden colony [phylotype garden] | 2 | 66 | 2.0147 | 0.1415 | |
| pupae colony [phylotype pupae] | 2 | 66 | 0.1314 | 0.8771 | |
| garden × pupae colony [phylotype garden,phylotype pu | 4 | 66 | 1.5345 | 0.2024 | |

**Table 5: Nested ANCOVA, PC3**

| ***Source*** | $df_n$ | $df_d$ | ***F*** | ***p*** | |
|---|---|---|---|---|---|
| Time | 2 | 66 | 0.8301 | 0.4405 | |
| phylotype garden | 1 | 66 | 1.5110 | 0.2233 | |
| phylotype pupae | 1 | 66 | 14.1206 | **0.0004** | Workers from Ps2 colonies spend more time allo-grooming |
| Time × phylotype garden | 2 | 66 | 1.7703 | 0.1783 | |
| Time × phylotype pupae | 2 | 66 | 0.6293 | 0.5361 | |
| phylotype garden × phylotype pupae | 1 | 66 | 0.2675 | 0.6067 | |
| Time × phylotype garden × phylotype pupae | 2 | 66 | 0.3091 | 0.7352 | |
| Median bacterial cover score | 1 | 66 | 3.5449 | 0.0641 | |
| garden colony [phylotype garden] | 2 | 66 | 1.4079 | 0.2519 | |
| pupae colony [phylotype pupae] | 2 | 66 | 1.7607 | 0.1799 | |
| garden × pupae colony [phylotype garden,phylotype pu | 4 | 66 | 3.4136 | 0.0134 | |

**Table 6: Nested ANCOVA, PC4**

| ***Source*** | $df_n$ | $df_d$ | ***F*** | ***p*** | |
|---|---|---|---|---|---|
| Time | 2 | 66 | 7.6289 | **0.0010** | Garden-weeding decreases over days |
| phylotype garden | 1 | 66 | 0.1954 | 0.6599 | |
| phylotype pupae | 1 | 66 | 0.1113 | 0.7398 | |
| Time × phylotype garden | 2 | 66 | 0.2100 | 0.8111 | |
| Time × phylotype pupae | 2 | 66 | 0.0030 | 0.9970 | |
| phylotype garden × phylotype pupae | 1 | 66 | 3.9861 | 0.0500 | |
| Time × phylotype garden × phylotype pupae | 2 | 66 | 0.1182 | 0.8887 | |
| Median bacterial cover score | 1 | 66 | 0.5304 | 0.4690 | |
| garden colony [phylotype garden] | 2 | 66 | 2.3617 | 0.1022 | |
| pupae colony [phylotype pupae] | 2 | 66 | 2.4638 | 0.0929 | |
| garden × pupae colony [phylotype garden,phylotype pu | 4 | 66 | 3.4072 | 0.0136 | |

**Table 7**: Correlations matrix between the performance of 9 behaviours, excluding three subcolonies that suffered significant garden losses at the time of fungus garden infection experiment. Product-moment correlation coefficients are shown, with signifcant correlations ($p<0.05$) shown in bold.

| | Garden-grooming | Weeding | Immobile inside fungus garden | Allo-grooming | Carrying eggs/larvae/pupae | Tending eggs/larvae/pupae | Trophallaxis | Manuring |
|---|---|---|---|---|---|---|---|---|
| Self-grooming | **-0.253** | 0.049 | **-0.560** | -0.140 | -0.157 | 0.079 | -0.013 | -0.136 |
| Garden-grooming | | -0.174 | **-0.312** | -0.141 | -0.088 | -0.004 | -0.178 | -0.001 |
| Weeding | | | **-0.289** | **-0.265** | 0.032 | 0.130 | -0.030 | 0.082 |
| Immobile inside fungus garden | | | | -0.080 | -0.138 | **-0.335** | -0.021 | 0.002 |
| Allo-grooming | | | | | 0.105 | **-0.305** | 0.091 | 0.071 |
| Carrying eggs/larvae/pupae | | | | | | 0.001 | -0.011 | -0.003 |
| Tending eggs/larvae/pupae | | | | | | | -0.020 | 0.013 |
| Trophallaxis | | | | | | | | -0.003 |

**Table 8: Binomial Generalized Linear Model, Proportion of time spent outside the fungus garden**

| ***Source*** | ***df*** | ***L-R*** $\chi^2$ | ***p*** | ***notes*** |
|---|---|---|---|---|
| Time | 2 | 7.047 | **0.0289** | Increase over days |
| phylotype garden | 1 | 3.732 | 0.0526 | |
| phylotype pupae | 1 | 13.194 | **0.0003** | Workers with Ps2 as native strain spend more time outside |
| Time × phylotype garden | 2 | 0.264 | 0.8781 | |
| Time × phylotype pupae | 2 | 3.070 | 0.2137 | |
| phylotype garden × phylotype pupae | 1 | 7.044 | **0.0078** | Workers with Ps2 as native strain spend less time outside in a Ps1 garden but more than ants with Ps1 as native strain |
| Time × phylotype garden × phylotype pupae | 2 | 0.458 | 0.7964 | |
| Median bacterial cover score | 1 | 2.368 | 0.1228 | |
| garden colony [phylotype garden] | 2 | 13.690 | **0.0010** | Caused by exclusion of colonies with garden-loss |

| | | | | |
|---|---|---|---|---|
| pupae colony [phylotype pupae] | 2 | 4.943 | 0.0832 | |
| garden × pupae colony [phylotype garden,phylotype pu | 4 | 26.321 | **<.0001** | Workers from Ae.150 in garden from Ae.160 spend more time outside; workers from Ae.160 in garden from Ae.150 spend less time outside |
