# Supplementary figures and images for: Interaction specificity between leaf-cutting ants and vertically transmitted Pseudonocardia bacteria
Source: BMC Evol Biol. 2015 Feb 25;15:27. doi: 10.1186/s12862-015-0308-2 (PMC4346108; doi:10.1186/s12862-015-0308-2)

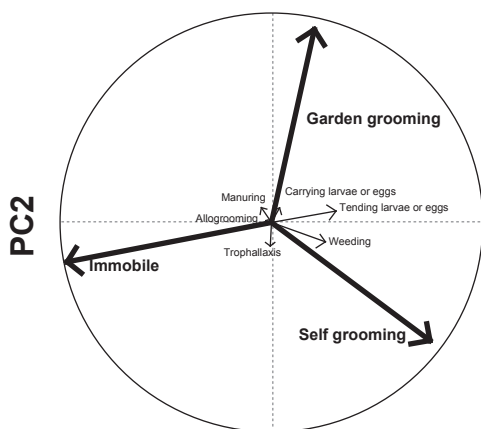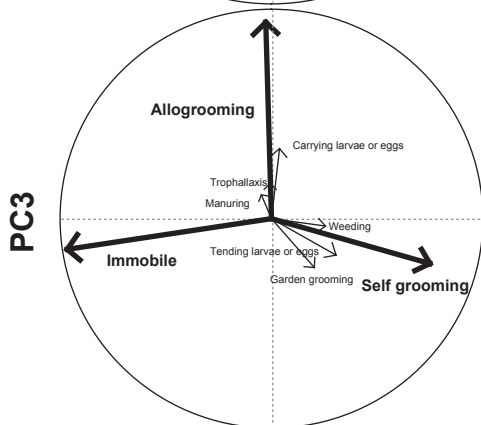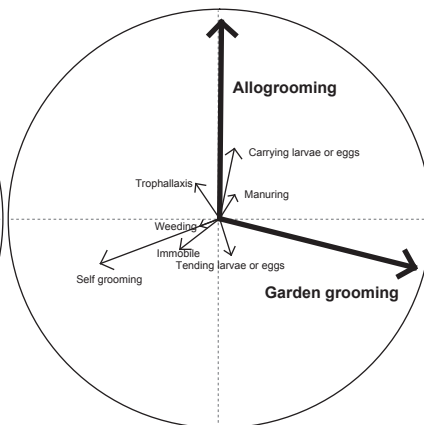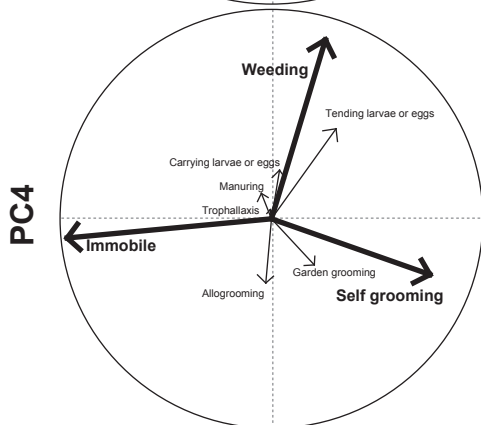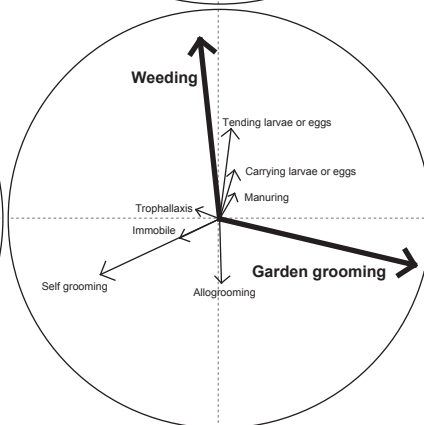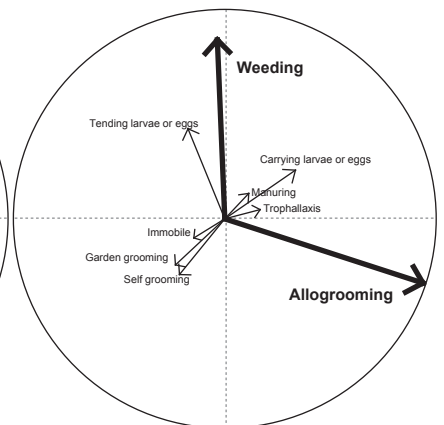

**PC1**

**PC2**

**PC3**

Supplement: Additional file 2: Figure S1. — Loading plots of the first four principal components (PC1-4) from a principal component analysis of the nine behaviors monitored within subcolony fungus gardens of our cross-fostering experiment. In each plot, the loading of particular components of behavior is plotted on a scale ranging from −1 to 1. Behaviors with the highest loadings for each PC are shown in bold, with the imputation that variation in those behaviors is well-captured by the principal component. [file 12862_2015_308_MOESM2_ESM.pdf]
